# Mitochondria dysfunction in circulating tumor cells

**DOI:** 10.3389/fonc.2022.947479

**Published:** 2022-08-04

**Authors:** Chiara Agnoletto, Stefano Volinia

**Affiliations:** ^1^ Rete Oncologica Veneta (ROV), Veneto Institute of Oncology IOV-IRCCS, Padua, Italy; ^2^ Laboratorio per le Tecnologie delle Terapie Avanzate (LTTA), Department of Translational Medicine, University of Ferrara, Ferrara, Italy; ^3^ Biological and Chemical Research Centre (CNBCh UW), University of Warsaw, Warsaw, Poland; ^4^ Center of New Technologies, University of Warsaw, Warsaw, Poland

**Keywords:** CTC, mitochondria, ROS, drug resistance, invasiveness

## Abstract

Circulating tumor cells (CTCs) represent a subset of heterogeneous cells, which, once released from a tumor site, have the potential to give rise to metastasis in secondary sites. Recent research focused on the attempt to detect and characterize these rare cells in the circulation, and advancements in defining their molecular profile have been reported in diverse tumor species, with potential implications for clinical applications. Of note, metabolic alterations, involving mitochondria, have been implicated in the metastatic process, as key determinants in the transition of tumor cells to a mesenchymal or stemness-like phenotype, in drug resistance, and in induction of apoptosis. This review aimed to briefly analyse the most recent knowledge relative to mitochondria dysfunction in CTCs, and to envision implications of altered mitochondria in CTCs for a potential utility in clinics.

## Liquid biopsy for tumor diagnostics

Cancer metastasis occurs through a series of sequential steps, which include epithelial mesenchymal transition (EMT) of primary tumor cells into tumor-initiating cells (TICs) and their intravasation into the bloodstream as circulating tumor cells (1), and subsequent extravasation at distant sites, with generation of metastasis upon mesenchymal epithelial transition (MET) (2–4). CTCs have been detected in the majority of patients with tumors, they have been proven to be heterogeneous and a subgroup of them represents cancer stem-like cells (CSLCs) or TICs (5, 6).

Recent progress in the identification of cancer biomarkers opened a new field of cancer diagnostics (7). Due to the non-invasive nature of liquid biopsy, repeated sampling and testing of blood have been performed for the accurate early disease detection and monitoring of treatment responses (8). In clinics, enumeration and phenotyping of CTCs have been proven as novel biomarkers to estimate the risk for metastatic relapse or disease progression in various tumors (9). CTCs have been detected in 30-50% of patients with metastatic colon, prostate and/or breast cancer (9–11). High CTC levels, as enumerated with the CellSearch™ assay, which specifically detects tumour cells with epithelial phenotype, defined as 4’,6-diamidino-2-phenylindole (DAPI)+, CK+, CD45- cells, are associated with poor clinical outcome, i.e. shorter progression free survival (PFS) and overall survival (OS), in breast, colorectal, and prostate cancers (3, 9, 10, 12–18). Advanced cancer patients with high CTC counts even after systemic therapy have poor clinical outcome, and elevation of CTC levels during follow-up predicts a high risk of progression (10, 19–21). The prognostic potential of CTCs for monitoring metastasis or the efficacy of chemotherapy has been reported: in metastatic breast cancer, persistently elevated CTC levels after one cycle of treatment correlates with a poor prognosis (9), while a reduction in CTC levels indicates improved prognosis (22–24).

Further, detection of CTCs might represent an alternative approach for early diagnosis (25). Technical advances have assessed the feasibility of detecting and profiling CTCs at the very early steps of tumor invasion (26). Indeed, CTCs have been detected in 10% of CRC precancers (adenomas) and in 3% of patients with chronic obstructive pulmonary disease (COPD), who have an elevated risk of developing lung cancer 1–4 years before CT screening of lung nodules (27, 28). In this contest, tissue-specific transcriptome profiling of single CTCs might address in verifying the location of the occult lesion, to select the appropriate imaging or diagnostic methodology (29).

As an alternative liquid biopsy, cell-free (cf) circulating DNA is continuously released by clonal tumor cells into the circulation (25). Alterations in cfDNA can be identified through ultra-deep NGS even at very low frequency (<1%), and have been used for early detection of recurrence in several tumors (e.g. colorectal cancer, pancreatic cancer, neuroblastoma) and, recently, also of premalignant lung and bladder disorders (27, 30, 31). At present, different platform technologies provide sufficient sensitivity in circulating tumor (ct) DNA detection to identify lung tumor patients relapsing within a year of subclonal detection, and for precision screening in cervical premalignancy (31, 32). However, methodologies require to be improved for detection and profiling of ctDNA, and measurements need higher sensitivity and specificity, by defining quantitative thresholds to avoid overdiagnosis. A relevant biological limitation is the amount of ctDNA recovered from early-stage cancer patients, even if less than 0.1% of ctDNA in plasma has been detected by digital droplet PCR or NGS methods (33). Enrichment steps have to be performed, based on biological properties or physics. Confounding results due to cancer-associated mutations not restricted to tumor patients and the presence of clonal alterations in blood cells due to aging and clonal hematopoiesis both represent critical issues (34, 35). Genomic driver alterations of tumors in BRAF, RAS, EGFR, HER2, FGFR3, PIK3CA, TP53, CDKN2A, and NF1/2 genes can also be identified in non tumor specimens. Additionally, determining the tissue of origin of the neoplastic lesion can be extremely challenging (27). Thus, limitations for clinical utility of ctDNA analysis are evident.

## Mitochondria dysfunction in cancer: minimal integrity point

An altered metabolism is a distinct feature of tumorigenesis, and the metabolic profile in tumour cells represents a crucial step for their survival. Tumor cells depend strongly on both enhanced glycolysis, the pentose phosphate pathway, and glutaminolysis, as a result of dysfunctional mitochondria, to efficiently respond to energetics requirements (36–39). Yet, tumour cells require a minimal functional mitochondrial pool, in order to produce sufficient amount of biosynthetic precursors (40–42).

Mitochondria act at multiple levels and coordinate several biological processes, with generation of reactive oxygen species (ROS), release of oncoproteins and oncometabolites, modulation of calcium homeostasis and autophagic processes, cell death, and metabolism (43, 44). Mitochondria integrity is a central checkpoint for cancer cells (36), as they actively participate in plasticity of tumour cells and act on several mechanisms to address environmental conditions.

Despite the mitochondrion constitutes a key actor, the dualities of its function in tumour metastasis and therapy resistance have only recently been depicted, with opposite effects on both processes. Through the analysis of large-scale data sets from The Cancer Genome Atlas (TCGA), the underpinning genetic determinants of these changes have been identified, which are orchestrated by oncogenes and tumor suppressors (45). Further, tumors share a subset of metabolic gene signatures independent of their tissue of origin, and upregulate genes that encode for glycolysis and nucleotide biosynthesis enzymes, with important implications for cancer diagnosis and patient stratification (46, 47). Experimental data suggest that mitochondrial dysfunction can reach a threshold where it turns to opposite effects for tumour cells, and a fine regulation of mitochondrial function is required to drive tumorigenesis. Coherently recent evidence indicates that the metabolic phenotype of cancer varies at different disease stages, and contribute to tumor progression (48, 49). Thus, stage-specific metabolic traits have been identified in prostate, breast, renal, and lung tumors (50–53), and transcriptional analysis of 21 tumor types collected by the TCGA confirmed the strict association of inhibition in genes of mitochondrial metabolism with the presence of an EMT gene signature, which is linked to tumor initiation, invasion, and metastasis, and poor clinical outcome (54). In accordance, complementary studies have confirmed that mutations of enzymes from the TCA cycle, SDH and FH, are linked to EMT and invasive phenotype in pheochromocytoma and paraganglioma and renal cancer (55, 56). Similarly, decreased mtDNA content, which is associated with bioenergetics defects, linked with poor patient prognosis in several tumours (57).

Yet, defects in mitochondrial function have been reported to reduce tumour aggressiveness, as demonstrated in renal oncocytoma, with an aberrant accumulation of dysfunctional mitochondria inhibiting the autophagic machinery (58). Consistent, inhibition of autophagy leads to mitochondrial dysfunction and reprograms tumor fate toward benign neoplasms (59).

The hypothesis is that the reduction in mitochondrial function could be progressively advantageous for tumour cells until a ‘minimal integrity point’, below which this alteration becomes deleteriuos.

The determinants of the metabolic adaptations during dissemination and metastasis are only partially defined. Overall, cancer cells that detach from the primary tumor experience oxidative stress, and thus activate mitochondrial antioxidant networks to eventually metastasize. Coherently, in human specimens of prostate cancer the peroxisome proliferator- activated receptor gamma coactivator 1 alpha (PGC1α), which is the master transcriptional regulator of mitochondrial oxidative metabolism, is downmodulated (50). The role of mitochondrial dysfunction in promoting metastasis is further confirmed by a partial inhibition of mitochondrial respiratory chain due to rotenone, with induction of cell migration and clonogenicity *in vitro* and lung metastasis *in vivo (* 60). Finally, mtDNA mutations affecting complex I support breast cancer metastasis *in vivo via* deregulation of NAD+/NADH and activation of autophagy (61).

Despite these consistent lines of evidence, an increased mitochondrial oxidative phosphorylation genes function was detected in CTCs from orthotopically implanted breast cancer mice, while in distant metastases expression of PGC1α was increased, suggesting that tumor-specific reprogramming might occur during metastasis, thus reconciling apparent discrepant data from literature (62). Of note, differential use of pyruvate in the mitochondria has been recently demonstrated to dictate the site of metastasis in breast cancer (63, 64).

Last, several results support a role for metabolic adaptation specifically mediated by activated mitochondrial function as a key determinant of therapy resistance (48). As an explicative example, resistance to mitogen-activated protein kinase (MAPK) inhibitors in BRaf^V600^-driven melanoma is associated with increased mtDNA content and oxidative phosphorylation (65), while inhibition of BRaf^V600^ induces an oxidative phosphorylation switch activated by PGC1α (66, 67), enhancing the detoxification capacities of these cell.

To date, numerous drugs have been proposed to modulate different functions of mitochondria for tumour therapy, which have been reviewed elsewhere (68, 69). Briefly, these strategies aim to compensate alterations in all relevant mitochondrial activities, i.e. bioenergetics, signaling, and biosynthetic functions (68, 69). At present, most drugs have been tested for antitumour activity in clinical trials (68). Moreover, the strict interconnection between mitochondrial metabolism and core cellular checkpoints reveals the potential of targeting mitochondrial activity in combination therapies (48). However, the variety of therapeutic targets and the ability of cells to adapt and compensate need to be considered. Mitochondrial metabolism is heterogeneous within and between tumors (70), as evidenced in the diverse responsiveness to antiangiogenic therapies (71–73), suggesting that the efficacy of anticancer therapy may depend on the adaptive metabolic capacity of tumour cells. Furthermore, cancer-initiating and therapy-resistant cells present a more oxidative metabolic program, thus the emergence of therapy-resistant cancer clones could rely on the newly acquired metabolic state, while this metabolic plasticity can be therapeutically exploited through the combination of standard and antimetabolic therapies (48).

In this article, our objective is to review the most recent evidence in support of a role for mitochondria dysfunction in circulating tumor cells behaviour, with the attempt to eventually reconcile apparent discordant results, and envision their implications for a potential utility in clinics.

## Potential impact of mtDNA mutations in CTCs

Mitochondrial DNA (mtDNA), with its mutations and polymorphisms, has only recently acquired novel attention in tumor research. Yet, mitochondrial genetics in cancer has been neglected for a long time. Only recent large-scale sequencing efforts and clinical studies have highlighted the prevalence of mutations in mtDNA and their potential roles in tumorigenesis (74, 75). Human mtDNA is maternally inherited, with several mtDNA copies per mitochondrion and hundreds of mitochondria per cell, and encodes 37 genes, which include 22 transfer RNAs, 2 ribosomal RNAs and 13 protein subunits of the electron transport chain (ETC) complexes and ATP synthase (mtOXPHOS proteins) (75, 76). mtDNA is highly polymorphic due to a mutation rate an order of magnitude higher than the nuclear genome. Further, functional variants can be beneficial or deleterious depending on the context. A subset of mtDNA variants have been reported to cause minimal adaptive changes in OXPHOS, with modulation of multiple mitochondrial functions including stress, autophagy, and oncogenic responses to environment (77–79). Moreover, mtDNA can also influence the inflammasome, innate immunity, IL-1b and NFkB inflammatory pathways, and T-cell immune surveillance (79, 80).

Several examples in literature reported that a nearly total loss of mtDNA copy number *in vitro* and *in vivo* results in subtle or temporally delayed effects on mitochondrial function (81). mtDNA is subjected to the phenomenon of heteroplasmy, i.e. the existence of diverse subsets of mtDNA molecules into a given cell, due to the multi-copy nature of mtDNA. Mitochondrial DNA heterogeneity occurs frequently and is an important concept for the development of mitochondrial dysfunction (82). The availability of great datasets, such as the International Cancer Genome Consortium (ICGC) and the TCGA, demonstrated that ~60% of all solid tumors present at least one mtDNA mutation (83, 84). A great majority of mutations are at high levels of heteroplasmy, with a minority of tumours achieving near-complete mutation homoplasmy, thus indicating that dysregulation of mitochondrial function *via* mtDNA mutation is a feature of tumour. Also, in general, oncocytic tumours with high heteroplasmy of mtDNA mutations, and significant mitochondrial dysfunction, are benign, non-aggressive, low proliferating lesions (85). Recent clinical and genetic studies pointed to mtDNA mutations as potential drivers or phenotypic modifiers of prostate and thyroid cancers (86, 87), yet a definitive experimental evidence of mtDNA mutations as a key driver event in tumorigenesis is lacking.

The heteroplasmic mtDNA genotype is continuously remodelled during successive cytokinesis, thus several genotypes with diverse oncogenic potential are generated among tissues within the same individual over time (77).

The effect of mtDNA haplotype in tumor predisposition and development has only recently been confirmed, as discussed in details in a recent review on the importance of mtDNA alterations to drive precision prevention trials (88–91). Inherited missense alterations, potentially extremely deleterious, in mtDNA genes, such as ND6 (NADH dehydrogenase subunit 6) and COI (cytochrome oxidase subunit I), which code for subunits of OXPHOS complexes I and IV, have been associated with risks of tumors (77, 78, 92–95). Such alterations in mtDNA are heteroplasmic and frequently lethal if exceeding a biochemical threshold, depending on several criteria, among which the type of tissue (93–96). In mitosis milder mtDNA polymorphisms can shift to become predominantly enriched within individual cells, potentially contributing to neoplastic transformation. The importance of this phenomenon for cancer predisposition has been demonstrated as the mtDNA complex I ND5 m.12425delA frameshift mutation, inherited as a germline mutation and transmitted at lower heteroplasmy levels (5–10% mutant), shifted to homoplasmic mutation exclusively in nasopharyngeal tumor cells and correlated with lack of the ND6 subunit (94). Genetic or pharmacologic (metformin) disruption of mitochondrial respiration increased autophagy and prevented cancer development in a mouse model of Li-Fraumeni syndrome. On the other hand, in a pilot study of Li-Fraumeni patients, metformin decreased mitochondrial activity while activating a cell-signaling event which is known to lead to rhabdomyosarcoma development (97). Of great relevance for clinical applications, nuclear DNA germline mutations influence mitochondrial genomic instability for cancer predisposition, as described e.g. for the nuclear genes BRCA1, SUV3, SOD (36, 74, 88, 98). Accumulating evidence suggests that mtDNA mutations may also contribute to cancer cell development, tissue invasion and metastasis. Indeed mtDNA variations, such as deletions, point mutations and copy number differences, are associated with several cancer types (99). In breast cancer, a compromised mitochondrial function, due to mtDNA mutations and low mtDNA copy number, has been associated with increased metastasis and poor prognosis (78, 100); also low mtDNA copy number promotes metastasis by inducing EMT *via* mitochondrial retrograde signaling (101). In addition, cells with compromised mitochondrial integrity rapidly progress to malignancy (74, 99), and clonal expansion of mutant mtDNA species was reported in 27–80% (average 54%) of malignant tumor samples (102).

Despite this evidence, it has been reported that mitochondria of tumor cells are functional and perform oxidative phosphorylation. This concept further supports the notion of a minimal integrity point determining the relevance of a defective mitochondrial function on tumorigenesis. As a proof, targeted depletion of mitochondrial DNA can reduce tumorigenic potential *in vivo (* 36). A recent paper demonstrated the effects of complete mitochondrial DNA deletion on the ability of tumors to metastasize *in vivo (* 40). Normal cells contain both discrete and networked mitochondria each with multiple mtDNA copies. Melanoma and breast carcinoma cells completely deprived of mtDNA, named ρ0 cells, upon injection intravenously in syngeneic murine models, have delayed tumor growth (40, 41). Of note, cells derived from primary tumors originating from ρ0 cells, and their circulating and metastatic counterparts, acquired a partial mitochondrial network, and progressively recovered a full respiratory function; this effect was associated with stepwise assembly of mitochondrial electron transport chain complexes and correlated with tumorigenicity (40). The acquisition of a full mitochondrial competence is dependent from horizontal mtDNA transfer, consistent with previous *in vitro* results (103, 104). A crucial step of full respiration recovery is associated with the assembly of the respirasome and ETC complex II (CII), in accordance with the requirement for efficient OXPHOS in metastatic dissemination (62). Consistent with these observations, autophagy is activated in CTCs when respiration is partially restored, in order to eliminate dysfunctional mitochondria. In accordance, higher levels of TFAM, a critical factor for replication, transcription, and packaging of mtDNA (105) and OPA1 (106) have been observed, while mitochondria-to-nucleus retrograde signaling eventually restores both mtDNA distribution and respiratory function (40). A minimum level of mtDNA damage is needed to initiate intercellular transfer of functional mitochondria. An independent confirmation has been reported in human glioblastoma cells (107). Thus, tumor cells deprived of mtDNA can acquire mtDNA of host origin, resulting in stepwise recovery of respiration from primary to metastatic tumor cells, with the crucial role of the complete assembly of the respirasome and CII (40).

As previously outlined, polymorphic sites are distributed along the complete mitochondrial genome. The genetic diversity of mtDNA in blood is strongly associated with tumor and may serve as a diagnostic marker. The small size (16,569 bp) of mtDNA is especially suitable for the accurate assessment of such profiles and the association of genetic heterogeneity, rather than specific mutations, with cancer, together with its clonal expansion, high copy number and high mutation rate (77). In a recent study, a significant depletion of mtDNA has been reported for several types of tumors, such as bladder, breast, kidney, and liver cancer (57), thus the identification of specific mutant variants in tested blood is quite difficult. Assessment of heterogeneity profiles of intra-host mtDNA variants from blood has been proven to overcome the identification of specific mutations for diagnostic detection of HCC (108). Consensus sequences of mtDNA differ between tumor and blood from ~ 58% of patients, while most tumor-specific variants (99.4%) were present in less than 5% of HCC patient, limiting their use as general cancer markers (108). In contrast, accurate estimation of heterogeneity can be performed at a moderate sequencing depth, thus providing a more reliable source of cancer-specific markers (108). Thus, a strong genetic signal consisting in a intra-host mtDNA profile has been documented, despite the presence of tumor-specific mutant mtDNA species at a very low concentration in plasma. However, at present the strict HCC specificity of the classifier has not been confirmed (108).

## Effect of dysfunctional mitochondria in CTCs

The deregulation of cellular energetics is a hallmark of tumor cells, with enhanced glycolysis, pentose phosphate pathway, and glutaminolysis, as a result of altered mitochondrial function (37, 38). In order to dissect the metastasis-related deregulation of metabolic genes in CTCs, a panel of genes have been analyzed in prostate cancer cell lines with different metastatic capacities (109). Eight metabolic genes were differentially expressed in metastatic cell lines, HK2, PDP2, G6PD, PGK1, PHKA1, PYGL, PDK1, and PKM2 (109), with a confirmed and remarkable association between their functions and the metastatic capacity of tumor cells (110–112). Of clinical relevance, the identified genes were detected in the CTCs of 54 clinical samples. Of note, two key enzymes of glycolysis and the pentose phosphate pathway, respectively, PGK1 and G6PD, were determined as efficacious markers for CTCs metabolic analysis (109). Further, PGK1/G6PD-marked hypermetabolic CTCs (GM+CTCs, i.e. DAPI+CD45−PGK1/G6PD+ cells) potentially represent a more accurate marker than EMT-CTCs for the diagnosis of metastasis in prostate cancer patients (109). Indeed, increased GM+CTCs level was associated with advanced tumor stage and metastasis (P < 0.05), and presented higher AUCs of the ROC curve (0.780) in the discrimination of metastatic patients than the EMT CTCs subtypes (E-CTCs 0.729, H-CTCs 0.741, and M-CTCs 0.648) (109).

In breast tumor patients CTC exhibited enhanced mitochondria biogenesis and respiration, with higher expression levels of genes associated with mitochondrial biogenesis (PGC-1α, PGC-1β, NRF1, and ERRα) and oxidative phosphorylation (Cox5b, Cox4i, ATPsynth, CytC) (62). CTCs were largely quiescent and specifically upregulated PGC-1α, and presented a more aerobic metabolism compared to both primary and metastatic tumors (62). These effects were proven to be mediated by PGC-1α (62). Of clinical relevance, high PGC-1α expression was detected in over 80% of CTC from IDC patients with lung metastases, confirming its association with distant metastasis and poor outcome (62). Thus, some invasive and migratory properties of tumor cells are dependent on mitochondrial respiration and PGC-1α is a potential target for therapeutic intervention. A dynamic shifts in the metabolic program of tumor cells facilitates diverse steps in cancer progression and metastasis, and mitochondrial biogenesis and respiration induced by PGC-1α is essential for functional motility of cancer cells and metastasis (62). The reversible shift in patterns of metabolic gene expression is synergistically coupled with genes frequently associated with EMT and acquisition of enhanced migratory and invasive properties of tumor cells (62). Several reports proved the mutual regulation of metabolic genes by EMT and vice versa, through both *in vivo* and *in vitro* experiments, thus synergistically promoting cancer metastasis (113–115). Altering mitochondrial function also determines survival and acquisition of cancer stem cell properties, in part *via* retrograde mitochondria-nucleus signaling (116). Mitochondrial activity and ROS detoxification are critical for cancer cell viability (117), and ensure cancer cell survival detaching from basement membrane (118). Further, E-cadherin expression is an important determinant of metastatic potential in metastatic lung nodules and CTCs in breast cancer (119), consistent with the loss of its expression due to EMT occurring frequently during tumor metastasis. E-cadherin activation inhibits metastasis at multiple stages, including the accumulation of CTCs from the primary tumor and the extravasation of tumor cells from the vasculature (119). Activating mAbs increased the frequency of apoptosis in CTCs and tumor cells in metastatic nodules, through upmodulation of Bax mRNA expression, and downmodulation of Bcl-xL mRNA expression (119). Overall these data reconcile with the notion that tumor-specific reprogramming might occur during sequential stage in tumorigenesis and in the metastatic cascade, as outlined previously in the manuscript (47, 48).

In a recent paper, testing the presence of CTC in peripheral blood of patients with renal cell carcinoma (RCC) undergoing surgery, authors reported a difference of the mitochondrial network between CTCs and basophils, monocytes and neutrophils, as evaluated by mitochondria staining, was observed (120). RCC is a highly invasive tumor, and patients respond poorly to chemotherapy, even in combination with immunotherapy (121, 122). Further, early detection of RCC remains a significant challenge. Predictive markers of response still lack in clinical practice. Testing CTCs profiles by gene expression analysis of the targetable genes may improve RCC therapy outcomes. Multiregional sequencing of RCC tumors and metastatic tissues evidenced the high intra-tumoral heterogeneity with respect to adjacent normal kidney tissue (123). The frequent lack of epithelial antigens and concomitant EMT in RCC tumor cells (124) often compromise CTC capture. The size-based isolation of CTCs by using the ISET (isolation by size of epithelial tumor cells) filtration method in combination with mitochondria staining allows to differentiate non-hematopoietic cells in the peripheral blood and define CTC subgroups possibly associated with metastatic potential, confirming that an altered mitochondrial network is relevant in determining the metastatic phenotype of CTCs (120). A relevant drawback in this study is the lack of data related to differences in mitochondrial network observed between CTCs and leukocytes, if related to mitochondrial volume or structure, and, especially, if a correlation with specific CTC subsets was conceivable.

## ROS function in circulation-related stresses in CTCs

Epithelial–mesenchymal transitioned CTCs enter into the vasculature, due to loose mosaic vessels and remodelling of extracellular matrix (ECM) (125, 126). Intravascularly, these metastasis-initiating CTCs need to maintain survival under anoikis, immune attack, and severe shear stress. Although the great majority of CTCs die into the circulation (127), 0.1% of CTCs survive as disseminated cells and eventually relapse (128). Into the circulation tumor cells respond to mechanical forces, and the role of the fluid microenvironment in metastasis has been recently proven, both as interstitial flow (~ 0.1 dyn/cm2), blood (1–30 dyn/cm2)/lymphatic circulation (~ 0.64 dyn/cm2), and target organ-specific fluid microenvironments (129). Of clinical relevance, the interstitial flow determines the direction of tumor cell metastasis to specific organs (130). One of the most crucial mechanical forces is generated by liquid flowing on the cell surface, i.e. the laminar shear stress (LSS) (129, 131). The laminar shear stress regulates the survival and function of normal cells, such as endothelial cells, osteoblasts, and circulating hematopoietic embryonic stem cells (132–135), and also promotes metastatic potential and anoikis resistance in breast CTCs (136, 137).

*In vivo* and *in vitro* evidence has proven that conversion from epithelial tumor cells into CSLCs/TICs can occur within blood vessels, due exclusively to hydrodynamic shear stress experienced during systemic circulation, without additional requirement for growth factors or a hypoxic stromal niche (138). A recent study has demonstrated that the fluid shear stress (0.05 dyne/cm2) in the interstitium can promote cancer motility through modulating the Yes-associated protein (YAP1)-related ROCK-LIMK-cofilin signaling pathway (139).

Adhesion to the ECM helps to maintain normal tissue architecture, and loss of anchorage activate a programmed cell death termed ‘anoikis’ (140). Anoikis is due to cell detachment from the ECM and prevents anchorage-independent tumor cell growth. CTCs need to acquire resistance to anoikis to survive in the circulatory system, where cells encounter the fluid shear stress. Resistance to anoikis is a hallmark of malignant tumor cells, and both the dynamic ECM network (141, 142) and hypoxic conditions (143) have been proven to promote anoikis resistance and increase survival in epithelial and carcinoma cells. Dissecting the molecular mechanisms that protect tumor cells from undergoing anoikis is critical, and novel strategies to target CTCs within the circulation could reduce their metastatic potential. Cancer cells acquire anoikis resistance *via* several mechanisms and signaling molecules including phosphoinositide 3‐kinase (PI3K)– protein kinase B (Akt) (144), Ras–extracellular signal regulated kinases (ERK) (145), Jun‐ N‐terminal kinase (146), mitogen‐activated protein–extracellular signal‐regulated kinase (147), and integrins (148). In addition, tumor microenvironments can also contribute to anoikis resistance in cancer cells by altering matrix rigidity, increasing oxidative stress, and depriving cells of adequate oxygen supply (149–151). An increased ROS level has been reported in CTCs (118). ROS have been confirmed to be crucial regulators of cell adhesion (8), and attachment of CTCs to the lining of the microvasculature is a crucial step for cancer cell extravasation and metastasis generation (152, 153). A high ROS level is associated with enhanced invasiveness and metastasis in hepatocellular carcinoma (HCC) (154). In pancreatic cancer and melanoma, acquisition of anoikis resistance protects cells from apoptosis, and promotes cell invasion and metastatic potential through the phosphorylation of STAT3 at Tyr705 (155). Anoikis is a highly complex multistep process, and both acquisition of apoptosis resistance and autophagy promote epithelial cell survival during anoikis (156).

Signaling activated by EMT‐related transcription factors constitutively activate specific signals in metastasis, including evasion of anoikis, with enhanced stemness and clonogenic features of cancer cells (157). The importance of EMT in metastasis is doubtful and recently discussed in several studies (158–160). Even the most mesenchymal states are not irreversibly committed (161), and a lot of studies support the notion that the metastatic potential greatly correlates with an intermediate EMT state (54, 125, 162–164). Yet, distinct hybrid phenotypes states determine the invasive, metastatic, and differentiation characteristics of tumor cells, with implications in tumor heterogeneity, invasion, metastasis, and resistance to therapy. Fluid shear stress experienced in systemic circulation can lead to specific acquisition of MSC-like potential in breast tumor CTCs, that promotes EMT, and acquisition of CSLCs/TIC potential (138). Such effects were proven to be mediated by promoting conversion of CD24middle/CD44high/CD133middle/CXCR4low/ALDH1low primary patient epithelial tumor cells into CD24low/CD44low/CD133high/CXCR4high/ALDH1high cancer stem-like cells (CSLCs), with plasticity and self-renewal capacity (138). This activation is dependent on ROS/NO generation, and suppression of extracellular signal-related kinase (ERK)/glycogen synthase kinase (GSK) 3β, an analogous mechanism operating in embryonic stem cells to prevent their differentiation while promoting self-renewal (138). Briefly, activated stress-responsive signaling pathways induces the transition from tumor cells to more highly invasive TIC (138).

Tumor cells detached from the ECM, upon invasion through the basement membrane, and entering into the circulatory system, encounter blood flow‐induced low shear stress (LSS; 2 dyn/cm2), which induces expression of Caveolin‐1 (Cav‐1), a 22‐kDa integral membrane protein. Cav‐1 has been proven to induce breast cancer cell motility, invadopodia formation, and metastasis *via* the PI3K–Akt–mechanistic target of rapamycin signaling pathway (141). In addition, LSS protects breast tumor cells from anoikis under anchorage‐ independent conditions *via* a Cav‐1‐ dependent signaling pathway, by inhibiting Cav‐1‐dependent extrinsic and intrinsic apoptotic crosstalk signaling (136). Indeed, LSS‐induced dissociation of Cav‐1–Fas inhibited the generation of the death‐inducing signaling complex, caspase‐8 activation, with inhibition of the extrinsic apoptosis signaling pathway (136). Likewise, LSS blocked the mitochondrial pathway through promotion of integrin β1–focal adhesion kinase‐mediated multicellular aggregation, suppression of truncated BID translocation, inactivation of caspase‐8 and mediated crosstalk between the extrinsic and intrinsic apoptotic pathways, which in turn inactivate Bcl‐2 and Bcl‐xL, thus preventing mitochondrial membrane permeabilization through Bax oligomerization (136). Accordingly, depletion of Cav‐1 restored sensitivity to anoikis. Also, upon LSS a significant decrease in Beclin‐1 has been observed, thus autophagy might be another regulator of LSS‐induced anoikis resistance (136). These data underline a novel role for flow‐induced shear stress in the regulation of anoikis in neoplastic cells, indicating that LSS‐induced anoikis resistance is a critical mechanism that increases tumor malignancy (136). In human lung carcinoma cells, ROS prevent Cav‐1 ubiquitination and degeneration (149). Also, a recent study demonstrated that Cav‐1 is involved in anoikis resistance in human lung cancer cells through regulation of myeloid cell leukemia 1 (Mcl‐1) by interacting with Mcl‐1 and preventing it from degradation (165). Overall, these results may lead to potential therapeutic strategies targeting Cav-1 and modulating the tumor microenvironment (136).

The effect of cyclic laminar shear stress (LSS) has been recently studied *in vitro* on CTCs of colorectal tumor (CRC) (166). Suspended tumor cells with a CK8+/CD45−/DAPI+ phenotype actively responded to LSS by activating the expression of atonal bHLH transcription factor 8 (ATOH8), a fluid mechanosensor, with key roles in intravascular survival and metabolism plasticity (166). Molecules, with the capability of sensing and translating mechanical forces, can transform physical stimulation into biological signals (167). As a new LSS-response molecule, ATOH8 is induced by 10 dyn/cm2 LSS in endothelial cells, and is also involved in angiogenesis, skeletal muscle formation, and embryonic development (168–170). ATOH8 expression among tumors is heterogeneous, and its role as a tumor suppressor or tumor promoter is still controversial. ATOH8 could inhibit stem cell features of hepatocellular carcinoma cells (171) and malignant phenotypes of nasopharyngeal carcinoma (172), while promoting cell proliferation and inhibiting apoptosis in CRC cells (173). In CRC it exerts a tumor promoting effect and is associated with hematogenous metastasis and poor prognosis in patients (166). Specifically, ATOH8 was upregulated in CTCs *via* activation of VEGFR2/AKT signalling pathway mediated by LSS induced VEGF release *in vitro* and *in vivo (* 166). ATOH8 transcriptionally activated HK2-mediated glycolysis and inhibited cell death pathway in CTCs, thus mediating the intravascular survival of colorectal tumor cells in the circulation, and ultimately providing a novel potential target for the prevention and treatment of hematogenous metastasis in CRC (166, 174) Metabolism and cell survival are inextricably linked, and cancer cells can switch between different metabolic states to respond to adverse conditions such as metabolic stress, anoikis, and mechanical stress (131, 175). ATOH8 overexpression could promote CRC CTCs migration, invasion, anoikis resistance, facilitating CTC survival (166). HK2 is one of the key enzymes of glycolysis, participating in the regulation of cancer cell metabolism and death, and its overexpression is significantly positively correlated with CRC recurrence (176). HK2 can support cell survival *via* promoting glycolysis and reducing overabundant ROS or generating HK2-VDAC complex with inhibition of mitochondria-mediated apoptosis. As expected, in ATOH8-overexpressing CRC cells ROS level were down-regulated while mitochondrial HK2 was up-regulated (166). Briefly, a VEGF-VEGFR2-AKT signal axis in CRC m-CTCs contributes to the high expression of ATOH8 and ultimately promotes CTC survival in the fluid microenvironment upon LSS exposure (166).

## Mitochondria and drug resistance in CTCs

CTCs may respond differently to chemotherapies compared to primary tumor cells. CTCs isolated from advanced breast cancer patients are more resistant to the DNA-damaging and pro-apoptotic effects of chemotherapy than tumor cells attached to the ECM (177). Of note, the response of CTCs to chemotherapy has a prognostic significance. Apoptosis in CTCs correlates with systemic chemotherapeutic response and disease progression upon therapy (177).

Chemotherapy activates the intrinsic pathway of apoptosis (178, 179). The anti-apoptotic Bcl-2 proteins are expressed on the mitochondrial membrane and prevent apoptotic cell death upon directly binding to pro-death Bax and Bak. ROS both induce the mitochondrial anti-apoptotic proteins *via* activation of the transcription factors NF-kB, Nrf-2, and HIF-1a (180, 181), and reduce the expression of pro-apoptotic proteins *via* the ERK/MAPK and PI3K/Akt pathways (182). As a proof of evidence for therapeutic strategies, caspase 3 activation and Bcl-xL depletion are correlated with a decreased number of CTCs and metastasis (119).The interactions of certain Bcl-2 proteins occur at the BH3 domains (178), and BH3 profiling measures the relative interactions of pro- and anti-apoptotic proteins to determine whether a tumor cell is near the threshold to activate apoptosis through mitochondrial outer membrane permeabilization (178, 179). Coherently, BH3 profiling acts as a metabolic signature and can predict response to chemotherapy and resistance to targeted therapy (183, 184). For example, a tumor with high level of functional Bcl-xL, an anti-apoptotic protein, may be resistant to therapy and BH3 profiling should correlate with lack of treatment efficacy (179). A recent trail, registered at Clinicaltrials.gov. (NCT03223662) has been set with the primary objective to determine whether a metabolomic signature or BH3 profiling of pre-neoadjuvant tumor biopsy correlates with the outcome of pathological complete response (pCR) after neoadjuvant chemoradiotherapy for patients with esophageal adenocarcinoma or squamous cell carcinoma, to serve as a basis for precision-based, personalized strategies for future treatment (185). Mitochondrial priming is dynamic, therefore, its threshold for apoptosis can be decreased by selecting tumor specific therapies. Stratification of patients based on whether pCR occurs may identify metabolomic signatures associated with response. Furthermore, future trials will be based on altering the mitochondrial threshold for apoptosis to increase the susceptibility to standard therapeutics.

Progression into the cell cycle, CD40L-NF-kB–mediated Bcl-xL upregulation, downmodulation in the expression of the proapoptotic Bim, Bax, and Bak proteins (186) and ultimately a decrease in mitochondrial priming and drug resistance have been recently confirmed in Mantle cell lymphoma (MCL) by using an ex vivo model (187). Using BH3 profiling, the central role of microenvironment-dependent signaling has been confirmed, with sequestration of the BH3-only activator Bim by Bcl-xL proteins at the mitochondrial level (187). Further, anti-CD20 antibody OBN Obinutuzumab efficiently counteract overexpression of Bcl-xL through NF-kB inhibition and loss of mitochondrial priming and drug sensitivity (187); consistently, OBN has demonstrated promising clinical activity with increased progression-free survival (PFS) in combination with bendamustine (188). The combined use of ibrutinib, which mediates indirect Bcl-xL down-modulation upon BTK-dependent binding, and venetoclax may improve clinical responses with more efficiency and less toxicity than the current standard of care. At present, an ongoing Obinutuzumab, GDC-0199 Plus Ibrutinib in Relapsed/Refractory Mantle Cell Lymphoma Patients (OAsIs) Trial for MCL patients (OBN, ibrutinib, and venetoclax, www.nationalclinicaltrials.gov,#NCT02558816) has been designed to determine *in vivo* efficacy through a selective approach targeting the lymphoma niche (187).

Induction of apoptosis is a common effect of several drugs. B-cell lymphoma 2 (Bcl2) expression is associated with resistance to apoptosis, and might be a marker for relative therapeutic resistance. Serial apoptosis monitoring might provide insight into resistance to a therapeutic regime. Bcl-2 should thus represent a biomarker of biological and clinical interest (189–191). A pilot study has been performed on metastatic breast cancer (MBC) patients with the aim to estimate Bcl-2 expression and apoptosis in CTCs after initiation of a new therapy, in order to assess the therapeutic efficacy (192). At baseline, apoptosis inversely correlated with CTC number and modestly with Bcl-2 positive CTC. As expected, higher CTC levels at baseline or first follow-up were associated with worse prognosis (9) and provide novel observations for Bcl-2 expression and apoptosis in CTCs. After one cycle of therapy in patients with elevated CTC, higher levels of CTC apoptosis were associated with worse prognosis, while higher CTC-Bcl-2 levels correlated with decreased apoptosis and superior PFS, resembling that for patients without elevated CTC (192). Actually the cohort consisted of a relatively small number of patients. In a recent paper, in patients with ER positive breast cancer who received adjuvant endocrine therapy, Bcl-2 predicted favourable outcomes, although its presence has been associated with worse prognosis (193–195). Since some patients had ER positive disease while others were ER negative, and some received endocrine therapy while others received chemotherapy, the association of CTC-Bcl-2 with outcomes may be quite heterogeneous. In summary, the results from this pilot study confirmed the prognostic significance of CTC at baseline and first follow-up for patients with metastatic breast cancer (192, 196). Further studies are needed to incorporate these assays into larger, more definitive trials as well as standard clinical practice.

CTCs have been reported as prognostic in all stages of breast cancer. A novel strategy for the isolation and expression profiling of pure populations of CTCs based on immunomagnetic capture and fluorescence-activated cell sorting (IE/FACS) has been described (197). Unsupervised hierarchical clustering revealed that CTC profiles clustered with more aggressive subtypes of primary breast tumors, with downregulated apoptosis, the relative absence of immune-related signals and down-modulation of ribosomes (197), relative to peripheral blood, suggesting a relatively quiescent state in circulation (198). As expected, CTCs from MBC had significantly higher risk of recurrence scores than primary tumors (197).

Recently a higher expression of Bcl2 and reduced expression of acetyl coenzyme A carboxylase-1 (ACC1) in tumors associated with CTC clusters (CTCcls) positivity has been reported (199). CTCcls represent a unique subset with higher metastatic potential and resistance to chemotherapy compared to single CTCs (5, 200). Of clinical relevance, patients with early-stage/locally-advanced BC have higher median CTCcl counts compared to patients with metastatic tumor (201). Further, CTCcls have a clinical prognostic value both at baseline and after treatment in terms of PFS and OS in cancer patients (9, 202, 203). Of note, a CTCcl+ gene signature consisting of 54 upregulated genes has been identified, significantly associated with poor relapse-free survival (RFS) in 360 patients with basal-like BC advancements (199), confirming Bcl2 expression as a poor prognostic factor in triple negative breast cancer (TNBC) patients, especially in the absence of adjuvant therapy (204). Also, Bcl-2 is upmodulated in CTC of xenograft models of TNBC (199), and correlated with higher levels of adhesion molecules including E-selectin, ICAM-1, and VCAM-1 (205).

Of clinical relevance, despite a subset of CTC consists of circulating cancer stem cells with high tumorigenic potential (206), CTCs are resistant to chemotherapy through mechanisms different from those activated in CSCs (207, 208). Stress induced in CTC, upon loss of ECM attachment, determines either anoikis-associated apoptosis (209) or generation of elevated levels of ROS (118, 210), with mild DNA damage and pre-activation of DNA checkpoints (211), as recapitulated in *in vitro* experiments. As a result, the DNA damage repair is efficiently activated upon chemotherapy, while inhibiting ROS production dramatically reduces the efficiency of post-chemotherapy DNA damage repair. Different from CTCs, breast CSCs had lower levels of ROS as compared with non-CSCs (212). A DNA damage repair response in cancer cells has been confirmed to determine tumor resistance to several DNA-damaging therapies, including anthracyclines and platinums (213). ATM/Chk2 and ATR/Chk1, which are two major kinase signaling pathways involved in the canonical DNA damage response network, are pre-activated in CTCs, and cause cell cycle arrest (177). Activation of checkpoint kinases represents an important mechanism limiting chemotherapeutic efficacy (213, 214). Thus, several Chk1/Chk2 inhibitors, including XL-844, AZD7762, and PF00477736, which potentiate the effects of DNA-damaging therapies by abrogating DNA damage-induced cell cycle arrest, have entered clinical trials for cancer therapy in combination with chemotherapeutic drugs (215). In accordance, these agents sensitize resistant CTCs to chemotherapy *in vitro*, with reduction of the number of CTCs and inhibition of lung and liver metastasis in xenograft models (177).

Coherently, exposure to cytotoxic/oxidative stress mediates a switch of CTC to a less proliferative but more drug-resistant phenotype (216). CTC in women with advanced oestrogen receptor (ER)-positive/human epidermal growth factor receptor 2 (HER2)-negative breast cancer acquire a HER2-positive phenotype after multiple courses of therapy (217, 218). While primary breast cancer is highly sensitive to HER2-targeted therapy, the clinical significance of acquired HER2 heterogeneity in metastatic tumor has been only recently analyzed (216). Cultured CTC isolated from women with ER+/HER2− primary tumors, 84% of whom had acquired HER2 expression, consisted of discrete subpopulations: a more proliferative HER2+ CTC subset, not addicted to HER2, consistent with activation of multiple signalling pathways, and a HER2− CTC subset, resistant to cytotoxic chemotherapy, while sensitive to Notch inhibition, due to activation of Notch and DNA damage pathways (216). Treatment of HER2+ CTCs with low doses of docetaxel or induction of oxidative stress induced rapid shifts from HER2+ to HER2−, thus modulating the HER2+/HER2− interconversion (216). HER2+ and HER2− CTCs interconverted spontaneously, and had comparable tumor initiating potential (216). Simultaneous treatment with paclitaxel and Notch inhibitors determined suppression of tumorigenesis in orthotopic CTC-derived tumor models (216). Together, these results point to distinct interconverting phenotypes within CTC, contributing to progression of breast cancer and acquisition of drug resistance.

Last, a number of studies have highlighted the role of tumor microenvironment in promoting tumor metastasis (219). In addition to circulating tumor cells, increased levels of viable circulating endothelial cells (CEC) are also released from primary tumors in patients with progressive disease (220). In head and neck cancer patients tumor-associated CEC express significantly higher level of Bcl-2, that is directly correlated with metastatic status, since they co-migrated with tumor cells to lung (221). CECs expressing Bcl-2 in the patient blood samples might be originating from tumor microvasculature and their binding to tumor cells induces a marked increase in Src and FAK activation in tumor cells, with anchorage independent survival (222), inhibition of both apoptosis, through regulating Bim, and anoikis, through regulating BAD (223, 224). Endothelial cells overexpressing Bcl-2 (EC-Bcl-2) expressed significantly higher levels of E-selectin and exhibited enhanced tumor cell binding (205). In addition, tumor cells bound to EC-Bcl-2 showed significantly higher anoikis resistance that was mediated by the Src-FAK signaling pathway (205). Furthermore, SCID mice coinjected with tumor cells and EC-Bcl-2 showed significantly higher lung metastasis (205). These results demonstrated a novel role for tumor-associated endothelial cells in protecting tumor cells from anoikis and chaperoning the tumor cells to distal sites.

## Discussion

**Figure 1:**
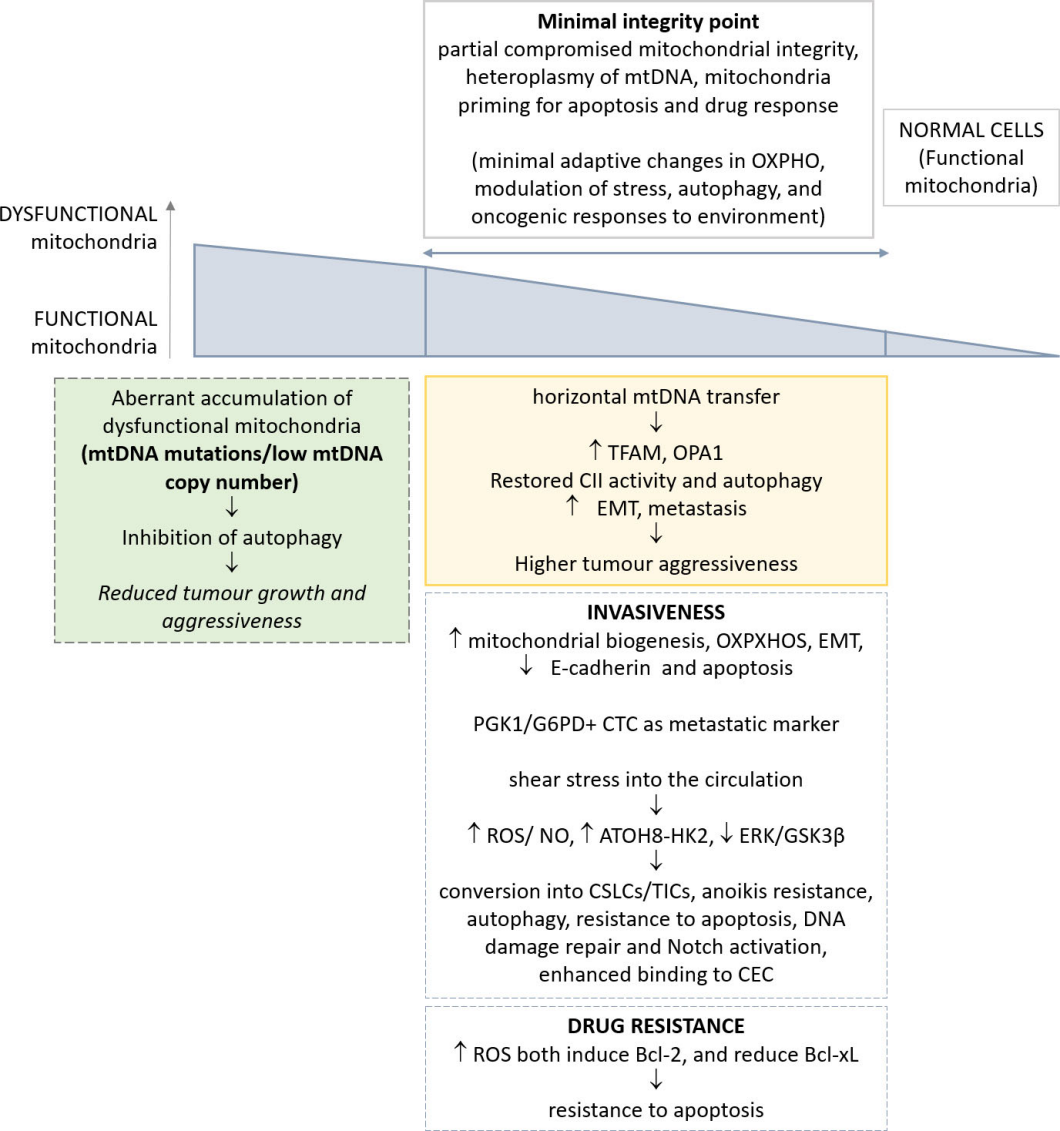
Schematic representation of the most relevant alterations reported in mitochondria, and their implications in CTC phenotype. The genetic and metabolic plasticity of tumour cells and the identification of a minimal integrity point threshold in mitochondria permit greater oncogenic/metastatic potential in CTCs. The effects of such adaptations in CTCs are depicted.

In the present review, we discussed the most recent research describing mitochondria alterations in the context of CTCs and their main effects on tumorigenesis and metastasis, and on CTCs behaviour, as summarised in **Figure 1** and **Table 1**. In literature there are relatively few data on this argument, with some main limitations related predominantly to the low detection rate and difficulties in isolation of viable CTCs from circulation.

**Table 1:** Most relevant data on the implications of mitochondria alterations identified in CTC, as discussed in the review.

Brief description of results	Preclinical models	Clinical samples	Reference	Year
Role of E-cadherin in accumulation, extravasation and lung metastasis generation of breast tumor cellsby inhibiting apoptosis	Mouse models of mammary cancer		(119)	2020
Ability to differentiate CTCs and leukocytes, and define CTC subgroups possibly associated with metastatic potential		Renal cell carcinoma (n = 186)	(120)	2020
Activation of ATOH8 expression in CTCs exposed to LSS, with induction of CTCs migration, invasion, anoikis resistance, survival; association of ATOH8 expression in CTCs with metastasis and poor prognosis in patients	Mouse models of colorectal cancer	Colorectal cancer (n=156)	(166)	2020
Acquisition of EMT properties and CSLCs/TIC-like potential in breast tumor CTCs upon fluid shear stress exposure	Cells from tumor tissues of patients with breast cancer		(138)	2019
Induction of Cav‐1 expression upon LSS, preventing anoikis through inhibition of apoptosis Decrease in Beclin‐1 upon LSS, with regulation of anoikis resistance through autophagy	Human breast carcinoma cells		(136)	2019
Identification of a CTCs gene signature associated with poor RFS in patients with basal-like BC advancements, confirming Bcl2 expression as a poor prognostic factor in TNBC	Triple-negative BC patient-derived xenograft transplantable models		(199)	2019
Detection of PGK1/G6PD+CTCs in 64.8% of prostate cancer patients, and association with advanced tumor stage and metastasis	Metastatic prostate cancer cell lines	Prostate tumor (n=54)	(109)	2018
HER2+ and HER2− CTCs interconverting subsets, with comparable tumor initiating potential, contributing to progression of breast cancer and acquisition of drug resistance	Orthotopic CTC-derived tumor models	ER+/HER2− breast cancer (n=19)	(216)	2016
Clustering of profiles of CTCs with more aggressive subtypes of primary breast tumors Higher risk of recurrence scores of CTCs from metastatic breast tumors than primary tumors		Metastatic breast cancer (n=5)	(197)	2015
Activation of autophagy, TFAM and OPA1, mitochondria-to-nucleus retrograde signaling in ρ0 CTCs with partially restored respiration	Mice model of metastatic melanoma and breast tumor ρ0 cells		(40)	2015
Higher resistance to chemotherapy in CTCs due to potentiated DNA repair, inhibition of checkpoint kinases Chk1 and Chk2 in CTCs to reduce cancer metastasis	Tumor xenografts	Metastatic breast cancer (n=60)	(177)	2015
Enhanced PGC-1α, mitochondria biogenesis and oxidative phosphorylation in CTCs from IDC patients with confirmed lung metastases and poor outcome	Orthotopically implanted breast cancer mice	Breast invasive ductal carcinoma (n=30)	(62)	2014
Association of higher CTC levels at baseline or first follow-up with worse prognosis Positive association of higher levels of CTC apoptosis with worse prognosis after therapy, and of higher levels of Bcl-2 positive CTC with decreased apoptosis and superior PFS		Metastatic breast cancer (n=83)	(192)	2013

Thus, most reports relate to a selection of tumor types, lacking a comprehensive overview of the significance of mitochondria dysfunctions in CTC and their role in mediating tumor metastasis. Despite this shortcut, a wealth of information still demonstrates a role for mitochondria in neoplastic transformation, and suggests a potential clinical use, for both diagnostic and therapeutic purposes.

A novel concept, outlined in the present manuscript, and supported by the most recent advancement on mitochondria dysfunction in tumor, is related with the high plasticity of these organelles. Altogether, the heteroplasmy of mtDNA, the identification of a minimal integrity point for mitochondria functionality, and, finally, the mitochondria priming in apoptosis and drug response influence the adaptive capability of these organelles to the requirement of cancer cells over time. Indeed, many apparently controversial reports from literature can be eventually reconciled, allowing for diverse tissues of origin and the different stages of cells throughout tumorigenesis and metastasis. Such adaptive responses of mitochondria render apparently difficult to depict a mode of intervention for their effective drug modulation.

Nevertheless, the interest in dissecting the mechanisms through which mitochondria might participate in determining CTC responses to microenvironment and dictating their metastatic potential, is obvious, both to the research community and clinicians. In consideration of the current evolution rate in CTCs isolation and profiling in a growing panel of tumors (e.g. mesenchymal versus epithelial tumors), we are expecting relevant advancement on this argument in the coming years. The possibility of detecting and more extensively characterizing mitochondria alterations in CTCs will allow to obtain more robust and direct proofs of the still incomplete data presented in this review.

## Author contributions

All authors participated in conceptualization, writing, review and editing the manuscript. All authors contributed to the article and approved the submitted version.

## Funding

The research was funded by Rete Oncologica Veneta (ROV).

## Conflict of interest

The authors declare that the research was conducted in the absence of any commercial or financial relationships that could be construed as a potential conflict of interest.

## Publisher’s note

All claims expressed in this article are solely those of the authors and do not necessarily represent those of their affiliated organizations, or those of the publisher, the editors and the reviewers. Any product that may be evaluated in this article, or claim that may be made by its manufacturer, is not guaranteed or endorsed by the publisher.
